# Evaluation of Yield Stability in Finger Millet (*Eleusine coracana* L.) Genotypes Using Multivariate Approaches

**DOI:** 10.1155/sci5/8628260

**Published:** 2025-11-02

**Authors:** Hailemariam Solomon Demissie, Chalachew Endalamaw Engida

**Affiliations:** ^1^Ethiopian Institute of Agricultural Research, Melkassa Agricultural Research Centre, Adama, Ethiopia; ^2^Department of Plant Sciences, University of the Free State, Bloemfontein, South Africa

**Keywords:** AMMI model, finger millet, GGE biplot, grain yield stability

## Abstract

Finger millet is commonly cultivated in the semiarid tropics, where it is primarily grown by subsistence farmers. However, grain yield remains low due to the complex quantitative nature of the trait and its low heritability. Therefore, genotype × environment interaction (GEI) significantly influences yield production. This study investigates the impact of GEI on the performance of finger millet genotypes across multiple environments, emphasizing the crop's sensitivity to climate variability. The objectives of this study were to evaluate the effects of genotype, environment, and GEI on yield and identifying high-yielding stable genotypes. Multienvironment trials (METs) were conducted at Axum, Negele Arsi, and Assosa during the 2018 and 2019 in summer cropping seasons utilizing row-column designs and advanced statistical analyses, including additive main effects and multiplicative interaction (AMMI) and genotype and genotype × environment interaction (GGE) biplot analyses. AMMI analysis indicated substantial environmental effects, with interaction principal component axes accounting for over 80% of the GEI. The GGE biplot identified the relationships between environments, highlighting specific genotypes that are optimal for each environment. Genotype G_32,_ with a yield of 2.75-ton ha^−1^, showed the highest mean yield values and the highest stability metrics using mean ranks and cultivar superiority stability values of 0.12 and of 9.0, respectively. Genotype G_53_ was the most stable, with a variance of ranks of 17.60, mean absolute difference of pairs of ranks of 4.90, and Wricke's ecovalence of 0.02. The choice of stability measures is critical, depending on plant breeders' objectives and the heritable traits targeted. Hence, genotype G_32_ had the highest grain yield performance and the most stable genotype and recommended for wider production in finger millet growing areas. Finally, the study demonstrates that AMMI and GGE are effective methods for selecting superior genotypes in diverse environments, providing valuable insights for finger millet breeding programs.

## 1. Introduction

Finger millet (*Eleusine coracana* subsp. coracana, 2*n* = 4*x* = 36) is one of the major cereal crops grown in tropical and subtropical Africa and India. It belongs to the family Poaceae and the subfamily *Chloridoideae*. Ethiopia is the world's second-largest producer of finger millet after India, with an annual production of 1.2 million tons per annum [1]. The crop is predominantly produced by subsistence farmers utilizing traditional varieties for human food and animal feeds [1]. Despite its inherent drought tolerance, finger millet productivity faces significant challenges from abiotic stresses, including temperature fluctuation, rainfall variability, soil conditions, and pest infestation [2, 3]. Exacerbating these issues is the prevalence of low yielding varieties and the impact of recurrent drought, which severely restricts grain yield potential [1]. To address these challenges and improve potential of finger millet, breeding programs should focus on maximizing grain yield, enhanced drought tolerance, and improving nutrient content [1, 4].

In finger millet breeding program, the former approach was to select genotypes based on just comparing the average productivity in their growing circumstances. Nevertheless, the crop's productivity is now greatly impacted by the genotype × environment interaction (GEI), and more data regarding the GEI in the breeding system should be produced before recommending genotypes for wider production [5]. This necessitates a thorough identification of genetically superior genotypes, particularly in the context of GEI, which significantly influences crop performance across diverse locations [6]. Furthermore, grain yield is a complex trait with a low rate of inheritance, and it needs a favorable environment to express its genetic potential [7]. The trait is significantly influenced by the GEIs and hinders the accuracy of yield estimation and reduces the association between genotypic and phenotypic values [8, 9].

Ethiopia has diverse agroecologies for the production of finger millets, with different distributions and patterns of rainfall, temperature, humidity, and elevation of the growing environment [10]. The Ethiopian Agricultural Authority has released and documented more than 27 improved finger millet cultivars for various agroecologies of Ethiopia [11]. The fact that each of these finger millet varieties reacts differently to the growing condition makes it impossible to understand the genotypes' performances and stability in a single season of trial testing. Genotype evaluation based on the grain yield at single location and year is inadequate to evaluate the crop's genetic potential because it ignores the impact of the interaction between genotype and environment [12]. This highlights the significance of GEI for the identification of superior genotypes and to ultimately to design a breeding strategy for the improvement of productivity [13–15].

The primary objective in crop breeding is to consistently increase grain yield, which significantly contributes to enhance productivity. This requires taking in to account the genotypes and the growing conditions which include rainfall, temperature, soil type, and humidity [12]. This implies that the crop improvement approach should be focused on minimizing the negative impact of the environmental effects, sustaining the genotypes for grain yield, reducing the cost, and increasing the production of the genotypes [16]. Since the environmental situation varies for the genotype performances, breeders should consider on how to select stable genotypes in the presence of GEI [17].

Several multivariate techniques, including principal component analysis (PCA), genotype plus GE interaction biplot (GGE) analysis, and additive main effects and multiplicative interactions (AMMI), have been employed for the investigation of GEI [18, 19]. AMMI is an essential model for assessing genotype performance that takes into account the sum of several multiplicative effects [20]. It also utilized to find discriminating genotypes with high mean yield and low interaction scores across a variety of environmental circumstances. The information generated from the AMMI is vital in breeding program to develop genotypes that is well adapted to the growing environments [21]. The PCA is used to generate biplots which help to identify the association between genotypes, environments, and GEI. This analysis has contribution to identify superior and stable genotypes in terms of grain yield for the targeted growing environments [22]. The principal component axis (PCA) scores and AMMI stability values (ASVs) are used to assess the genotypes' stability across locations [23]. For each genotype, the ASV is based on the IPCA1 and IPCA2 (interaction principal components axes 1 and 2, respectively) scores from the AMMI model [24]. The genotypes with the lowest ASV are regarded as the most widely adapted. Likewise, more stability is indicated by an IPCA2 score close to zero, whereas more responsive and less stable genotypes are represented by large values.

The GGE analysis used to investigate two significant sources of variation: the genotype main effect and the GEI of multienvironment trial (MET) data [18, 25]. When assessing genotypes, GGE biplot analysis assumes that only the G and GE effects are significant and should be taken into account at the same time. Because of this, the GGE biplot has been used in crop variety trials to evaluate the yield and stability of genotypes, determine the best genotypes for mega-environment delineation, and effectively identify the genotypes that perform the best across environments [26, 27].

The two models (AMMI and GGE) are essential for plant breeders to identify the performance and stability of genotypes in mega-environment trials. However, it is vital to consider the variations between the two models, where the AMMI model is used for AMMIs [28], and the analysis is based on double-centered PCA [29]. On the other hand, the GGE is for genotype main effects plus GEI [30], and it is based on environment-centered PCA [27]. In general, understanding and mitigating the effects of the GEI are crucial for ensuring accurate yield estimation and maximizing the correlation between genotypic and phenotypic values, ultimately contributing to enhanced and sustainable finger millet production. Therefore, this study aimed to evaluate the magnitude of genotype, environment, and GEI effects on grain yield in finger millet. This study assessed the stability and performance of genotypes across diverse environments using AMMI and GGE biplot models to identify high-yielding, stable genotypes suitable for specific mega-environments within Ethiopia's finger millet production areas.

## 2. Materials and Methods

### 2.1. Plant Materials and Experimental Sites

**Figure 1:**
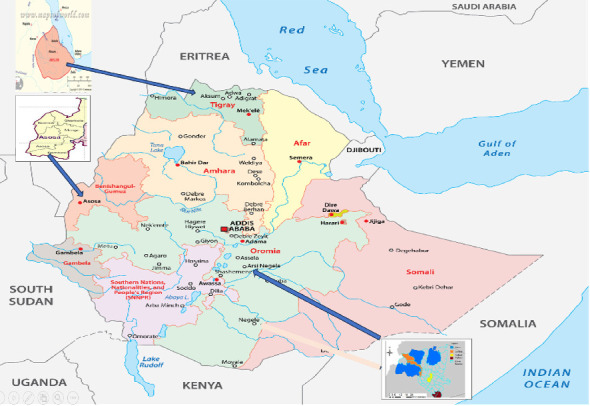
Geographical descriptions of the study locations.

A total of 55 elite finger millet genotypes including one check variety (Tessema) were evaluated for their genetic performances in major finger millet growing areas. The materials were introduced from the International Crops Research Institute for the Semi-Arid Tropics (ICRISAT) and evaluated in the national breeding system across multiple environments to assess their performance and stability at different locations. Accordingly, the genotypes were evaluated for two consecutive years (2018–2019) at Axum and Negele Arsi and for one year (2019) at the Assosa due to a shortage of seed in 2018. The three sites are important finger millet-producing areas in Ethiopia and provide suitable environments for evaluating genotype performance (Figure 1). Previous studies have described the rainfall distribution in these areas. Axum, located in northern Ethiopia, experiences a unique meteorological climate influenced by its elevation and geographical position. Most of the precipitation occurs during the main rainy season from June to September, with a secondary rainy period in March and April. Negele Arsi, in the central Oromia region, receives abundant rainfall mainly between July and September. Assosa, in western Ethiopia, also receives sufficient rainfall; however, its agricultural productivity is often constrained by soil moisture conditions [31, 32]. The details of the meteorological and soil property information are described in Table 1.

### 2.2. Experimental Designs and Agronomic Practices

Row-column designs with three replications were applied at three locations over two consecutive years. Each genotype was planted in a 5 m plot length, and the seeds were sown in three rows per plot. The row and plant spacing were 0.40 m and 0.15 m, respectively. Seeds were sown manually by drilling by hand, and then, the seedlings were thinned to have the recommended population density per hectare of land. Inorganic fertilizer of DAP at the rate of 100 kg ha^−1^ was applied at planting time while UREA at the rate of 50 kg ha^−1^ at planting and tillering stages, respectively. Field management practices such as weeding, thinning, insect pest control, and similar agronomic practices were applied uniformly at all locations.

### 2.3. Phenotypic Data Collection

Plant and plot-based data were collected across environments. Days to flowering was recorded as the number of days from emergence to the stage when ears emerged from 50% of the tillers per plot. Plant height (cm) was recorded by measuring the height of plants from ground level to the tip of inflorescence (ear) at the dough stage. Finger length (cm) was recorded from the base of the ear to the tip of the finger at each of the five randomly taken plants of main tillers at the dough stage. Fingers number per ear was recorded from five randomly taken plants at harvest. The number of productive tillers per plant was counted from five randomly taken plants of each plot at harvest. Grain yield was determined by harvesting all plants from the three rows of each plot. The seeds were weighed by a sensitive balance and adjusted to 10% moisture content by drying in the sun.

### 2.4. Data Analysis

The phenotypic data were collected from each location, and the combined mean analysis was analyzed using SAS software Version 9.1 [33]. Homogeneity of residual variance was determined by Bartlett's homogeneity test. The site regression model which is sometimes called the GGE model was used to analyze GEI, which is a powerful tool for effective analysis and interpretation of data from METs. To explore G plus GE variability in grain yield of finger millet, the SREG model was used as presented by the following equation [18]:

(1) $$\begin{array}{c} Y_{ij\text{⁣}}=\mu +\beta_{j\text{⁣}}+\overset{k}{\underset{n=1}{\sum }}\lambda_{\text{in}}\;\xi_{\text{in}}\;\eta_{\text{in}}+\varepsilon_{ij}, \end{array}$$

where *Y*_*ij*⁣_ is the mean of genotype *i* in environment *j*; *μ* is the grand mean; *β*_*j*⁣_ is the environment *j* main effect; *n* is the singular value; *λ*_in_ and *ξ*_in_ are the singular vectors for genotype and environment for *n* = 1, 2,…, respectively; and *ε*_*ij*_ is the residual effect.

### 2.5. Stability Analysis

Several multivariate approaches have been used to investigate GEI, including PCA, AMMI, and genotype plus GEI biplot analysis. AMMI isolates genotype and environmental main effects and applies interaction PCA to explain patterns in the GEI or residual matrix, yielding a multiplicative model. The AMMI biplot analysis was an effective tool to diagnose the GEI patterns graphically. The AMMI model combined analysis of variance (ANOVA) for the main effects of the genotype and environment with PCA of GEI and used PCA for visualizing the GEI in what is known as a biplot diagram [19, 34]. The biplot display of PCA scores plotted against each other provided visual inspection and interpretation of the GEI components. Integrating biplot display and genotypic stability statistics enables genotypes to be screened and selected in a plant breeding program. Based on this, AMMI model was used to determine the stability of the genotypes across environments using in GenStat software [35]. The AMMI model first fits the additive effects for the genotypes and the growing environments and the multiplicative term for GEIs. The AMMI model, according to Farshadfal [36], is presented as follows:

(2) $$\begin{array}{c} Y_{ij}=\mu +g_{i}+e_{j}+\overset{n}{\underset{k=1}{\sum }}\lambda_{k}\alpha_{jk}\gamma_{jk}+e_{ij}, \end{array}$$

where *Y*_*ij*_ is the yield of the *i*th genotype in the *j*th environment; *g*_*i*_ is the mean of the *i*th genotype minus the grand mean; *λ*_*k*_ is the square root of the eigenvalue of the PCA axis *k*; *α*_*jk*_ and *γ*_*jk*_ are the principal component scores for PCA axis *k* of the *i*th genotype and the *j*th environment, respectively; and *e*_*ij*_ is the residual. The environment and genotypic PCA scores are expressed as unit vector times the square root of *λ*_*k*_, i.e., environment PCA score = *λ*_*k*_^0.5^*Y*_*ik*_; 0.5; genotype PCA score = *λ*_*k*_^0.5^*α*_*ik*_.

ASV was calculated for each genotype according to the relative contributions of the principal component axis scores (IPCA1 and IPCA2) to the interaction sum of squares. The ASV as described by Purchase [24] was calculated using the following equation:

(3) $$\begin{array}{c} \text{ASV}=\sqrt{{\left[\frac{\text{IPCA}1_{\text{ sum⁣of⁣squares}}}{\text{IPCA}2_{\text{ sum⁣of⁣squares}}}\right]}^{2}\left(\text{IPCA}1_{\text{score}}\right)+{\left(\text{IPCA}2_{\text{score}}\right)}^{2}}, \end{array}$$

where IPCA1_Sum of squares_/IPCA2_Sum of squares_ is the weight given to the IPCA1 value by dividing the IPCA1 sum of squares by the IPCA2 sum of squares. The larger the IPCA score is, either negative or positive, the more adapted a genotype is to a certain environment. Smaller ASV scores indicate a more stable genotype across environments [36].

Yield stability (YS) index was introduced by Kang, and this statistic would help breeders in selecting the genotypes with high and relatively stable yields across different environments as it integrates stability and yield performance of genotypes. Hence, YS index was also calculated using the sum of the ranking based on yield and ranking based on the ASV:

(4) $$\begin{array}{c} \text{YSI}=\text{RASV}+\text{RY}, \end{array}$$

where RASV is the rank of the genotypes based on the ASV; RY is the rank of the genotypes based on yield across environments. YSI incorporates both mean yield and stability in a single criterion. Low values of both parameters show desirable genotypes with high mean yield and stability [37, 38].

### 2.6. Environmental Variance (*S*^2^)

Romer [39] proposed the variance of yield performance for test genotypes across environments as a stability parameter. The genotype grain yield variance is taken in to account in all tested environments. The mathematical equation for this parameter is as follows:

(5) $$\begin{array}{c} S^{2}=\sum \frac{{\left(R_{ij}-m_{i}\right)}^{2}}{\left(e-1\right)}\;, \end{array}$$

where *R*_*ij*_ is yield of *i*th genotype in the *j*th environment, *m*_*i*_ is grand mean yield across all environments, and *e* is the number of environments. The minimum value of *S*^2^ refers to the greatest stability. Derived stability measures include the square root value (S) and its coefficient of variation.

### 2.7. Mean Variance Component (*θ*)

Plaisted and Peterson [40] proposed the variance component of GEI for interactions between each of the possible pairs of genotypes. This statistic considered the average of the estimate for all combinations with a common genotype to be a measure of stability. This stability statistic is described by the following equation:

(6) θ=p2p−1q−1∑j−1qxij−X¯i.−X¯.j.+X¯…2+SSGE2p−1q−1,SSGE=∑∑xij−X¯i.c−X¯.j.+X¯…2.

In the above equation, *x*_*ij*_ is the grain yield of genotype *i*th in environment *j*th, ${\overset{¯}{X}}_{i.}$ is the mean grain yield of genotype *i*th, ${{\overset{¯}{X}}_{.j}}_{.}$ is the mean grain yield of the environment *j*th, ${\overset{¯}{X}}_{..}$ is the grand mean, SSGE is the GEI sum square, and *p* and *q* are the numbers of genotypes and environments, respectively. Based on this statistic, the genotypes that show a lower value for *θ*_*i*_ are considered more stable.

### 2.8. Wricke's Ecovalence (*W*^2^)

Using the dynamic concept of stability, Wricke's model is the simplest method to evaluate the stability. Wricke suggested the ecovalence (*W*^2^) concept as the ratio of the interaction sum of squares contributed by each genotype to the GEI sum of squares. In other words, the ecovalence of the *ith* genotype is its interaction with the environments, squared and summed across environments. Thus, genotypes with low values have smaller deviations from the mean across environments and are more stable [41]. Greatest stability is when *w*_*i*_^2^ = 0. The following equation shows the mathematical process of this stability statistic:

(7) $$\begin{array}{c} W^{2}=\sum {\left(x_{ij}--{\overset{¯}{X}}_{i.}-{{\overset{¯}{X}}_{.j}}_{.}+{\overset{¯}{X}}_{\ldots }\right)}^{2}, \end{array}$$

where *x*_*ij*_ is the grain yield of genotype *i*th in environment *j*th, ${\overset{¯}{X}}_{i.}$ is the mean grain yield of genotype *i*th, ${{\overset{¯}{X}}_{.j}}_{.}$ is the mean grain yield of the environment *j*th, and ${\overset{¯}{X}}_{..}$ is the grand mean.

### 2.9. Static Variance

The economic significance of stability for the cultivation of a genotype was first identified by Roemer who used the variance across environments as a parameter for YS. This stability parameter follows a biological/static sense, implicating that a stable genotype is recognized as the one having small variance across the tested environments [39]. Therefore, to estimate the static phenotype stability of the genotype, the following equation was used:

(8) $$\begin{array}{c} S_{i}^{2}=\frac{\sum {\left(X_{ij}-X_{i}\right)}^{2}}{E-1}, \end{array}$$

where *X*_*ij*_ is the performance of the *i*th genotype in the *j*th environment, *X*_*i*_ is the mean performance of the *i*th genotype, and *E* is the number of environments.

### 2.10. Components of Variances

Genotypic, phenotypic, environment, and genotype ^∗^ environment variances were estimated:

(9) $$\begin{array}{c} \delta_{p}^{2}=\left(\delta_{g}^{2}\right)+\left(\frac{\delta^{2}ge}{E}\right)+\left(\frac{\sigma_{e}^{2}}{ER}\right), \end{array}$$

where *δ*_*g*_^2^ is genotypic variance, *δ*_*p*_^2^ is phenotypic variance, *δ*^2^*ge* is genotype ∗ environment variance, *σ*_*e*_^2^ is pooled error, *E* is number of environments, and *R* is number of replications.

Genotypic coefficient of variation (GCV) and phenotypic coefficient of variation (PCV) were estimated according to Singh and Chaudhary [42] as follows:

(10) $$\begin{array}{c} GCV\;\left(\%\right)=\left(\frac{{\surd \delta }_{g}^{2}}{X}\right)*100\%, \end{array}$$

(11) $$\begin{array}{c} PCV\;\left(\%\right)=\left(\frac{{\surd \delta }_{p}^{2}}{X}\right)*100\%, \end{array}$$

where *δ*_*g*_^2^ is the genotypic variance, *δ*_*p*_^2^ is the phenotypic variance, and *X* is grand mean.

## 3. Results

### 3.1. ANOVA

The ANOVA results for finger millet genotypes are presented in Table 2. The ANOVA revealed that highly significant variations (*p* < 0.001) among the finger millet genotypes, the environments in which the genotypes were tested, and the GEI showed the highest sum square for grain yield. In addition, genotype ^∗^ location (environment) interaction effects were highly significant (*p* < 0.001) for DTF, PHT, NF, FL, and GY.

### 3.2. Genotype Mean Performances

Table 3 lists the finger millet genotypes' mean grain production and yield-related traits that were assessed in each of the five environments. The genotypes G_24_ and G_16_ showed the longest anthesis times, with 94.45 and 81.40 days for blooming, respectively. Genotype G_25_ showed the highest number of productive tillers (PT = 19), and the lowest number (3.57) was for genotype G_20_. Across the whole tested environment, the highest and the lowest mean values of grain yield were exhibited for genotype IEFX12005 (G_32_) (2.75 ton ha^−1^) and genotype IEFV 10017 (G_5_) (1.57 ton ha^−1^), respectively. The control variety (Tessema) produced 1.81 tons of grain per hectare, but genotype G_32_ had the highest grain production per unit area. This indicated that G_32_ was more productive than the check variety. Additionally, the first mean high-yielding genotype (G_32_) took fewer days (86.53 days) to flower as compared to others.

The performances of 55 finger millet genotypes at each tested environment are indicated in Table 4. Among the tested environments, Negele Arsi in 2019 recorded the highest average grain yield, while Negele Arsi in 2018 recorded the lowest. The genotype IEFX12005 (G_32_) had the maximum grain production in AN18 and AN19, with 2.178 and 4.88 tons per hectare, respectively. Furthermore, this genotype (G_32_) performed better in terms of grain yield than the standard check variety of G_55_ (Tessema). In a similar vein, G_51_ and G_15_ produced the highest grain at AX18 and AX19, yielding 2.36 and 2.27 tons per hectare, respectively. These two genotypes (G_51_ and G_15_) showed a consistent grain yield production at these two environments (AX18 and AX19). The experiment was conducted at Assosa for just one year, and G_45_ recorded the maximum grain yield (3.03 ton ha^−1^) in 2019 rainy season.

### 3.3. Estimation of the Variances

In Table 5, the ANOVAs for some key agronomic traits of finger millet genotypes were explained. The result indicated that there were highly significant variances (*p* < 0.001) for finger length and number of finger traits. However, the grain yield showed highly significant variation (*p* < 0.01) and nonsignificant variations for the plant height trait.

The ANOVA of the finger millet genotypes is described in Table 6. For the grain yield traits, the finger millet genotypes showed PCV and GCV of 35.04 and 26.07 percent, respectively. A finger length trait has a PCV of 27.31 and a GCV of 15.86%. However, the GCV and PCV for the number of fingers are 10.11% and 22.58%, respectively. With regard to the environmental variances, the grain yield trait had 39.36% of environmental coefficient of variance (ECV).

### 3.4. Stability Analysis

The performances of 55 finger millet genotypes were evaluated across locations and over years (i.e., location-year combinations), and the grain yield stability (YS) of the genotypes is indicated in Table 7. The most stable genotypes were determined using various stability measurement approaches for the genotypes that were evaluated in five distinct environments. Based on this, using cultivar superiority value stability statistics, G_32_ was the most stable genotype and exhibited the highest grain yield of 2.72 tons ha^−1^. Static stability was used to evaluate the genotypes' stability, and the control variety (G_55_) was the most stable, followed by genotypes G_30_ and G_43_, respectively. Finger millet genotypes G_53_ and G_12_ with low Wricke's ecovalence ratings produced more grain and were the most stable finger millet genotypes. Using mean ranks value coefficients, the stability of the grain yield of the finger millet genotypes was computed; the genotypes with the lowest mean ranks value were considered to be the most stable genotypes. Therefore, using mean rank value stability analysis, genotypes G_32_ and G_42_ were the first and the second most stable genotypes, respectively. Using the mean absolute difference of pairs of ranks (MADPR), genotypes G_53_ and G_12_ were found to be the most stable, while genotypes G_24_ and G_21_ were the least stable. Based on the variance ranks value, genotypes G_53_ and G_12_ were the two most stable genotypes, while genotypes G_24_ and G_21_ were the least stable genotypes. In general, the most stable genotypes of finger millet could generally be identified using various stability measurement types; therefore, G_53_ was the most stable genotype using the MADPR, variance of ranks value, and Wricke's ecovalence stability coefficients, while G_32_ was the most stable genotype by the mean ranks value and cultivar superiority value stability analysis.

### 3.5. AMMI Analysis

The AMMI analysis is the technique to analyze the two-way experimental data; whose main effects are additive and the interaction effect is multiplicative. AMMI is assuming that all effects (except error) are fixed. Based on this, the grain yield of finger millet genotypes was investigated by AMMI ANOVA (Table 8). The result of the analysis showed highly significant variations between genotypes, environments, interactions, IPCA 1, and IPCA 2 parts. The genotype and environment variance percentages were 10.08 and 12.76, respectively. Likewise, the percentage of G x *E* that represented the GEIs was 33.74. This result indicated that the effect of the environments was more significant on the performances of the genotypes for the grain yield trait.

### 3.6. AMMI Biplot Analysis

**Figure 2:**
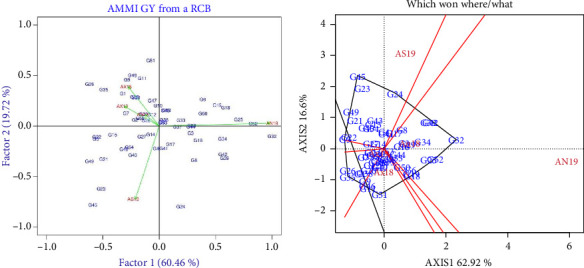
Genotype-by-environment interaction for grain yield of finger millet genotypes: (a) AMMI biplot of IPCA1 against IPCA2 and (b) GGE biplot identifying winning genotypes. AX19 = Axum in 2019; AX18 Axum in 2018; and AN18 = Negele Arsi in 2018; AN19 = Negele Arsi in 2019; Assosa in 2019.

The AMMI biplot analysis displays a graphic of the 55 finger millet genotypes in five distinct environments in Figure 2(a). The *x*-axis displays the main effects, and the *y*-axis displays the first PCA axis. The biplot showed diverse genotype responses to the study environments and accounted for 80.18% of the total treatment SS, leaving a substantial 19.82% in the residual. The graph showed the environmental scores, which are linked to the origin by a sideline. Of the five environments, AN19 had the longest line to the graph origin's center, while AN18 had the shortest line. The graph also indicated that the highest mean performance of the grain yield was exhibited by genotype G_32_, followed by G_52_ at the AN19 environment.

Similarly, the genotypes were dispersed across the graphs at various distances from the center of the graph's origin. The genotypes farthest away from the graphic's origin contributed most to increasing the GEI, for instance, genotypes G_32_, G_45_, and G_24_. On the other hand, the genotypes, for instance, G_53_, G_31_, and G_36_, contributed the least to the GEI, as they were closer to the center of the origin of the axes. Genotypes close to the tested environments had better grain yield performances. The genotype G_32_ had better grain yield performance in environment AN19. The two tested environments of AN19 and AS19 were the most discriminating, as indicated by the long distance between their marker and the origin. Closer relationships were observed for the environments of AX19, AX18, and AN18. The genotypes exhibited high mean grain yield in the environment AN19, and the remaining environments showed consistent performances. On the other hand, the environments of AN19 and AS19 contributed more to the interactions than the AN18 and AX19 environments, which contributed the least, and AX18 contributed moderately to the interaction effects.

### 3.7. GGE Biplot Analysis

GGE biplot analysis was used to evaluate the finger millet genotypes' performances and revealed the two most significant sources of variation: genotype main effects and GEIs. In Figure 2(b), the best genotype(s) in each environment and groups of environments have been indicated. The polygon is formed by considering the markers of the genotypes that are furthest away from the biplot origin, so that all other genotypes are contained in the polygon. As a result, the vertex genotypes such as G_32_, G_35_, G_51_, G_18_, G_32,_ and G_45_ had the longest vectors in their respective directions, which was a measure of responsiveness to the environments. Therefore, the vertex genotypes were among the most responsive genotypes; all others were less responsive in their respective direction. It is possible to compare the two adjacent vertex genotypes with a polygon. So, genotypes, for instance, G_52_, G_32_, and G_34_, had better grain yield performances in the environments of AN19 and AN18, but genotype G_32_ had superior performance in both environments.

**Figure 3:**
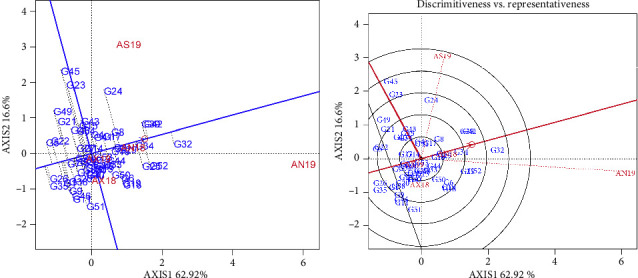
GGE biplots illustrating for finger millet genotypes (a) genotype mean performance and (b) comparison with the ideal genotype to assess performance and stability. AX19 = Axum in 2019; AX18 Axum in 2018; and AN18 = Negele Arsi in 2018; AN19 = Negele Arsi in 2019; Assosa in 2019.

The finger millet genotypes were assessed for mean grain yield performances through a biplot as indicated in Figure 3(a). An average tester coordinate (ATC) horizontal axis passed through the biplot origin and the average location, and the oval showed the positive end of the ATC horizontal axis. The average grain yield of the finger millet genotypes was estimated by projections of their markers onto the ATC horizontal axis. The genotypes were ranked based on their average grain yield performances for their respective tested environments. Projection of genotype markers onto this axis should, therefore, approximate the mean yield of the genotypes. Based on this, genotypes G_32_, G_42_, and G_39_ had the highest yield, and in contrast, genotypes G_35_ and G_21_ had the lowest grain yield. The discriminativeness and representativeness of the finger millet genotypes are indicated in Figure 3(b). The environments of AN19 and AX19 were ideal environments for the highest discriminating competence and the representativeness of the test environment. The ideal environments for the tested genotypes were compared using the angle with the average environment axis (AEA). So, the AN19 environment had a smaller angle with the AEA, while the AS19, AX19, and AX18 had larger angles with the AEA (Figure 2(b)).

**Figure 4:**
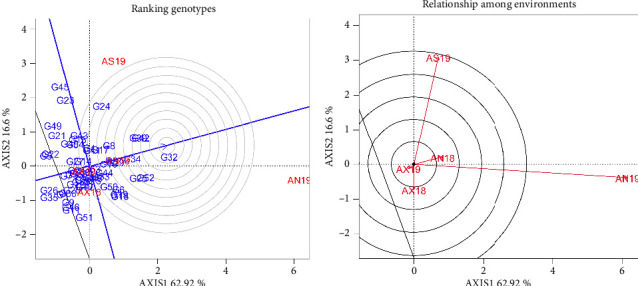
GGE biplot for grain yield of finger millet genotypes; (a) Ranking of the genotypes with reference to the ideal genotype and (b) the relationship among the environments. AX19 = Axum in 2019; AX18 Axum in 2018; AN18 = Negele Arsi in 2018; AN19 = Negele Arsi in 2019; Assosa in 2019.

The ranking of the genotypes with respect to the ideal environments is indicated in Figure 4(a). The center of the concentric circle in the GGE biplot showed an ideal genotype for the targeted environment. Among the genotypes, G_32_ was the most superior finger millet genotype, and the closer an environment was to this virtual ideal location, the better it was as a test environment. Therefore, AN18 was the best environment for the finger millet genotypes. This indicated that Negele Arsi was the most suitable environment for finger millet production. In contrast, the three environments (AN19 and AS19) were located far from each other, which elucidated that breeders should select genotypes for specific adaptation.

## 4. Ranking of Genotypes and Relationship of Environments

In order to select the best genotypes for wider cultivation, the finger millet genotypes were ranked according to their grain yield performances, as shown in Figure 4(a). The genotypes ranked in the first class were used to increase the productivity of finger millet with desirable traits, proving that G_32_ is the ideal genotype and that it exhibited superior grain yield across locations. Additionally, the analysis of the relationship between the tested locations was necessary due to the existence of the GEI in all of the tested environments.  Figure 4(b) shows the relationship between the tested environments for the performances of finger millet genotypes. The lowest angle existed between AN18 and AN19, which provided the existence of a high correlation between the two environments. On the other hand, a wider angle was noticed between the AX18 and AS19 locations.

## 5. Discussion

### 5.1. Variances of the Components

The genetic composition of the finger millet genotypes and how it interacts with its growing environments mostly determined its phenotypic expression. With appropriate parameters, the observed phenotypic variability would have a significant contribution for plant breeders to identify which traits of interest were heritable and nonheritable components [43]. Furthermore, the degree of genetic variability in finger millet genotypes would have an impact on the improvement of the crop, and this could be achieved through the use of biometrical techniques, such as GCV and PCV [44]. For the key agronomic parameters of the finger millet genotypes, the estimations of PCV values were greater than the GCV values, indicating that environmental influences had an impact on the traits (Table 6). On the other hand, this result confirmed the importance of the GEI, which is used to select suitable genotypes for the particular targeted environments. Additionally, the grain yield had a higher ECV value (39.36%), indicating that environmental factors had an impact on the genotypes of finger millet's performance in each of the studied locations.

### 5.2. Identification of Genotypes Using AMMI and GGE Biplot Analysis

The AMMI model contributed to the identification of finger millet genotypes that were evaluated across different environments. In the AMMI model, the *x*-axis displays the main effects and the *y*-axis displays the first PCA axis. The graph clearly explained that the environmental scores were connected to the origin by a side line (Figure 2(a)). The distance of the environmental line from the center of the origin illustrated the presence of the interaction force in the response of the genotypes. Based on this, environments with short lines to the center of the origin did not exert strong interaction forces, while those with long lines exerted strong interaction [45]. According to this principle, the AN18 environment exerted a lower interaction force than the AN19 and AS19 environments. Additionally, the graph demonstrated that genotypes that were similarly adapted to the targeted environments were grouped, and the environments that were grouped had a comparable effect on the genotype's performance [46]. As a result, genotypes of G_1_ and G_38_, for example, are situated between the environments of AX18 and AX19; they have a similar level of adaptation to these environments.

The genotypes were dispersed across the graphs at various distances from the center of the graph's origin, as indicated in Figure 1(a). The genotypes farthest away from the graphic's origin contributed most to increasing the GEI, for instance, genotypes G_32_, G_45_, and G_24_. Similar results were reported by Hossein in his AMMI study on sesame crops [43]. On the other hand, the genotypes, for instance, G_53_, G_31_, and G_36_, contributed the least to the GEI, as they were closer to the center of the origin of the axes. In other words, these genotypes had less response to the interaction and showed general adaptation to the test environments [47]. The environment that was more suitable for finger millet genotypes to express their genetic potential was identified; as a result, the highest mean grain yield was exhibited in environment AN19. Therefore, AMMI is the most powerful model to identify genotypes and environments that are well adapted to the targeted environments [48].

In order to determine the superior genotypes and optimal environments, as well as the genotypes' performance and stability, the finger millet genotypes were examined using the GGE model. Therefore, the vertex genotypes were among the most responsive genotypes; all others were less responsive in their respective direction (Figure 2(b)). The vertex genotypes within the polygon, such as G_32_, G_51_, G_35_, G_22_, and G_45_, are the most responsive genotypes to the environmental changes. The finger millet genotypes were ranked based on their average performance across the tested environments (Figure 4(a)). Based on this, G_32_ was the best-performing genotype and exhibited a consistently high yield across varying conditions. The ranking of genotypes is vital for breeders to select genotypes for further testing or commercial release. In general, among the tested finger millet genotypes, G_32_ was the most superior genotype that could be selected using the AMMI and GGE biplot models. This implies that both models are suitable for identifying superior genotypes, and similar results were reported by Mohammed [49].

### 5.3. GEI

The relationship of the tested locations in reference to the ideal genotypes was illustrated using the GGE biplot analysis (Figures 3(b) and 4(b)). In the AMMI method, the genotype that had the highest normal yield determined by the first PCA was the winner, and the normal yield was plotted against environmental PCA scores. In the GGE biplot, the which-won-where biplot was generated by joining the outermost genotypes to form a polygon within which all other genotypes lay inside as indicated in Figure 2(b). A perpendicular was drawn to the sides of the polygon through the origin, and the environments were divided into different sectors, each with a different vertex genotype. Each sector containing an environment was considered to demonstrate the relationship of the environments, with vertex entry as a winning genotype. Winning genotype is defined by high grain yield and minimum GEI for the given tested environment.

The genotypes of finger millet performed differently in each of the tested environments in terms of grain yield. This is because the GEI influenced the genotype's response that depends on external environmental factors. There were variations in the sensitivity of the finger millet genotype to the environments that were observed for the GEI. In the presence of the GEI, the breeder preferred a stable genotype with high yielders across the environments and minimum GEI [50]. To identify stable genotypes and investigate the GEI among crops, numerous statistical methods have been proposed by different scholars [51, 52]. However, for the multilocation trial evaluation, AMMI and GGE are the most powerful methods to identify stable genotypes and the identification of environments for optimal finger millet production [50, 53].

### 5.4. Identification of Stable Genotypes Using Various Stability Indices

Finger millet genotypes were evaluated at various locations and revealed highly significant differences between the genotypes, the environment under test, and the GEI. Due to the significance of the GEI, data were analyzed in detail using different statistical approaches prior to recommending a genotype for the targeted areas [54]. A range of stability metrics was used to calculate the finger millet genotypes' stability for grain yield as indicated in Table 7. However, there were stable genotypes but poor grain yield performances across the tested locations. This implies that unless a particular genotype exhibits superior grain production performance across all locations, stability alone is insufficient. This is the main task of the breeder: to identify suitable genotypes in changing environments [55].

The stability of finger millet genotypes was identified using AMMI methods, which have a significant contribution to determining the types of stability in finger millet genotypes, which has implications for designing breeding strategies. The finger millet genotypes were assessed using variety/genotype superiority values, which is one of the stability measurement methods that is widely used in crop improvement programs. In this type of stability measurement, the values of individual genotypes were calculated as the sum of the squares of the differences between their means in each tested environment. Finally, the mean of the best-performing genotype was divided by twice the number of environments [56]. Therefore, using cultivar superiority values, IEFX12005 (G_32_) was the most stable genotype in diverse environments (Table 7).

Plant breeders use another method of stability measurement, which is called static stability. The static stability of finger millet genotypes was assessed, which provides information on the ability of the genotypes to perform in the face of environmental fluctuations. Static stability genotype tends to maintain a consistent yield across the environments [57, 58]. Based on this, “Tessema” (G55) was used as a standard check and exhibited static stability across the environments. The variety exhibited a consistent grain yield across the tested environments. Nevertheless, it performed poorly in terms of grain yield under all environments. This type of stability is more beneficial in a variety of environments and is also referred to as biological stability [59]. Assessing genotypes with a static method, the genotype does not exhibit altered grain yield performance in response to environmental changes. The disadvantage of this approach is that genotypes with strong static stability tested under various environmental conditions exhibited low yield. As a result, plant breeders are not encouraged to use this technique to assess the yield of the genotypes or other associated random factors or their phenotypic stability. However, it is helpful to assess the phenotypic stability of traits that ought to hold steady. For instance, traits like disease resistance, drought, cold, and heat stress tolerance are among them [60]. Scholars outlined the advantages of static stability in the presence of GE influences that promote YS. However, it does not capture the genotype's potential for yield improvement across environments [15]. Therefore, plant breeders should have to consider the type of traits that are being used to improve the genotypes for the targeted environments.

On the other hand, the finger millet genotypes were assessed using a nonparametric method that includes the variance of genotypic rank values. Based on the variance of the ranks value, IEFX12001 (G_53_) was the most stable genotype. Likewise, the stability of finger millet genotypes was assessed using Wricke's ecovalence [41]. In this regard, stability measurement was the contribution of each genotype to the genotype-by-environment sum of squares in an unweighted analysis of the genotype-by-environment means. A low value indicates that the genotype responds consistently to the changes in the environment. Based on ecovalence stability, IEFX12001 (G_53_) was the most stable genotype. On the other hand, according to the mean ranks value, genotype IEFX12005 (G_32_) was the most stable genotype. In 2018 and 2019, this genotype performed best at the Negele Arsi location and had the highest mean grain yield performance. Better performance was also displayed by the genotype in the remaining test locations. A similar study was conducted by Khalil and Pour to identify higher adaptability of varieties of barley crops using the mean values of the genotypes, and such type of stability has contributed to getting more adaptable genotypes with high mean grain yield across the environments [61]. This type of stability is vital for plant breeders to improve crop grain yield performance and is found to be the best stability identification measure for finger millet genotypes. Similarly, the stability of the finger millet genotypes was computed using MADPR types of stability, and IEFX12001 (G_53_) was the most stable genotype. In general, the stability of finger millet genotypes was computed using different types of stability measurements as indicated above. Finger millet genotype selected using static stability measurement is not recommended because the genotypes had a consistent grain yield performance across the environments, but the grain yield was low. However, breeders can use this type of stability for the improvement of other traits related to disease and drought resistance breeding programs. The stability of grain yield was varied and measured at the level of the genotypes and also the combined or mean values of all genotypes in a given environment [62]. Therefore, the above-mentioned stability values can be summarized as genotype G_32_ being the most stable genotype in cultivar superiority value and in mean rank values. On the other hand, genotype G_53_ was the most stable genotype in ecovalence stability, MADPR, and variance of the ranks value stability metrics.

## 6. Conclusion and Future Perspectives

In conclusion, the ANOVA revealed highly significant variations among finger millet genotypes, environments, and GEIs for grain yield. Genotype G_32_ showed superior stability and consistently high yield across various environments, making it the most stable and high-performing genotype. The AMMI and GGE biplot analyses identified G_32_ as the best-performing genotype, demonstrating its adaptability to different environments and superior grain yield performance. Various stability measurement approaches consistently identified G_32_ as the most stable genotype, making it an ideal candidate for further breeding programs and commercial release. The findings underscore the importance of GEIs in optimizing finger millet production, suggesting that targeted breeding strategies can enhance YS and adaptability in diverse conditions. For future research, integrating AMMI and GGE models into breeding programs is encouraged to better exploit GEI and select stable, high-yielding genotypes. Further studies should explore multiyear and multilocation trials particularly at Assosa because the trail was conducted only for 1 year and suggested to conduct the trial to incorporate more diverse genotypes, and assess other traits such as nutritional quality and stress tolerance to enhance the adaptability and productivity of finger millet across varied agroecologies.

## Figures and Tables

**Table 1:** Description of the metrological and soil property information for the study areas.

Site	Metrology data						Soil properties		
	Temperature (°C)		Rainfall (mm/year)	Altitude (m.a.s.l)	Latitude	Longitude	PH	Organic carbon (%)	Soil texture
	Min	Max							
Axum	15	27	539–942	2000–2500	14°06′N	38°36′E	6.5	1.2	Loamy
Assosa	15	28	1128–1358	1401–1544	10°02′ N	34°32′E	5.6	1.4	Clay
Negele Arsi	10	25	500–1200	1500–3000	7°21′ N	38°42′E	7.5	1.37	Sandy loam

**Table 2:** Combined analysis of variance for finger millet genotypes evaluated at different environments.

Source of variation	DF	Sum of square					
		DTF	PHT	NF	FL	PT	GY
Genotype (G)	54	78.37^∗∗∗^	488.37^∗∗∗^	3.48^∗∗∗^	5.26^∗∗∗^	2.55^∗^	0.79^∗∗∗^
Replication (L)	6	60.37^∗∗^	1019.18^∗∗∗^	3.83^∗^	7.37^∗∗∗^	58.52^∗∗∗^	1.85^∗∗∗^
Location (L)	2	109865.83^∗∗∗^	117561.85^∗∗∗^	159.52^∗∗∗^	149.98^∗∗∗^	1794.17^∗∗∗^	3.49^∗∗∗^
Year (Y)	1	757.35^∗∗∗^	710.95^∗∗^	11.73^∗^	67.46^∗∗∗^	0.05ns	27.28^∗∗∗^
*G* × *L*	108	29.77^∗∗∗^	286.98^∗∗∗^	2.77^∗∗∗^	2.59^∗∗∗^	2.50^∗^	0.73^∗∗∗^
*L* × *Y*	1	3.94ns	710.95^∗∗^	11.73^∗^	67.46^∗∗∗^	0.05ns	20.04^∗∗∗^
*G* × *L* × Y	108	17.12^∗∗^	100.56ns	1.52ns	2.56^∗∗∗^	1.71ns	0.59^∗∗∗^
Residual	542	11.83	90.9117	1.56	1.05	1.65	0.32

*Note:* PHT, plant height.

Abbreviations: DF, degree of freedom; DTF, days to flowering; FL, finger length; GY, grain yield; NF, number of fingers; ns, nonsignificance; PT, productive tiller.

^∗^Significance at *p* = 0.05.

^∗∗^at < 0.05.

^∗∗∗^highly significant at < 0.001.

**Table 3:** Mean grain yield and yield-related traits of finger millet genotypes evaluated across the five environments.

Genotype	Code	DTF	PT	FL	NF	GY
IEFX12015	G1	90.470	5.040	6.900	6.190	1.72
IEFX12027	G2	93.330	5.690	5.370	6.850	1.85
IEFX12003	G3	86.330	4.550	6.690	6.910	2.15
IEFX12017	G4	91.930	4.760	6.520	6.590	1.97
IEFV 10017	G5	87.870	4.360	5.930	6.040	1.57
IEFX12035	G6	89.530	4.520	7.280	7.010	2.23
IEFV 10004	G7	90.200	4.800	5.830	5.840	1.76
4646	G8	90.270	4.710	6.720	6.690	2.05
IEFX12024	G9	88.930	4.650	6.490	6.240	1.81
IEFX12018	G10	90.330	4.830	6.630	6.280	1.86
IEFX12023	G11	88.200	4.710	6.360	6.790	1.73
IEFV 10006	G12	88.800	4.730	5.970	6.760	1.71
IEFX12022	G13	90.000	5.130	6.250	6.080	1.97
IEFX12011	G14	88.670	4.710	6.210	6.310	1.90
IEFV 10018	G15	88.600	4.360	6.330	5.930	2.20
KAT FM1	G16	81.400	3.910	6.110	6.170	2.00
IEFX12028	G17	91.670	5.010	6.680	6.050	2.11
IEFX12021	G18	91.730	5.490	6.200	6.790	1.99
IEFX12026	G19	90.670	4.810	5.860	6.190	2.09
IEFX12010	G20	91.400	3.570	6.950	6.720	1.80
6350	G21	93.000	5.600	6.070	6.730	1.76
IEFX12002	G22	90.800	4.250	6.390	5.560	1.69
3189	G23	91.870	4.830	4.930	7.690	1.98
2710	G24	94.450	5.810	5.830	6.440	1.98
Okhale 1	G25	89.400	19.000	6.810	7.030	2.32
IEFX12013	G26	87.200	4.730	7.170	6.400	1.77
IEFX12030	G27	88.930	4.990	6.310	6.090	1.80
IEFX12034	G28	93.400	5.810	6.100	6.120	1.85
IEFX12016	G29	89.530	4.720	7.950	7.800	2.25
IEFX12029	G30	89.670	4.910	5.770	6.680	1.96
5106	G31	89.670	4.560	5.870	6.030	1.71
IEFX12005	G32	86.530	5.210	6.570	6.450	2.72
IEFX12004	G33	89.670	5.310	7.670	6.760	2.01
KNE796	G34	87.870	4.710	6.570	6.490	2.33
IEFX12020	G35	87.930	5.030	6.090	6.240	1.61
IEFX12032	G36	91.070	4.640	6.330	5.930	1.78
IEFX12019	G37	88.200	4.570	6.610	6.560	1.81
IEFX12014	G38	91.600	4.970	7.150	7.400	1.72
3945	G39	87.400	5.000	6.000	6.710	2.45
IEFX12012	G40	91.400	5.190	6.610	6.760	1.86
IEFV 10028	G41	89.733	4.480	6.560	6.760	1.97
6473	G42	88.200	5.550	6.850	5.730	2.53
IEFV 10005	G43	90.450	5.370	6.410	6.320	2.00
IEFX12025	G44	91.330	5.550	6.070	7.640	1.99
IEFV 10027	G45	88.800	4.950	5.970	6.440	1.94
IEFX12031	G46	90.000	5.000	6.590	6.370	1.83
IEFX12007	G47	86.800	5.210	6.430	6.970	1.96
IEFX12008	G48	90.330	5.320	6.390	6.960	2.02
6059	G49	90.870	4.610	7.120	6.710	1.81
IEFV 10021	G50	87.400	5.080	7.500	6.480	1.98
IEFX12009	G51	91.000	4.570	6.000	6.000	1.97
IEFX12033	G52	89.200	5.310	6.670	6.950	2.34
IEFX12001	G53	89.230	5.810	6.790	6.480	1.99
IEFX12036	G54	93.530	5.680	8.040	6.000	1.93
Tessema (check)	G55	93.230	4.810	7.390	7.150	1.81
Mean		89.820	5.190	6.488	6.532	1.96
LSD		3.485	1.044	1.156	1.054	0.46
CV		3.830	25.583	15.860	19.118	20.00

Abbreviations: CV (%), coefficient of variation; DTF, days to flowering; FL, finger length; GY, grain yield; LSD, least significance difference; NF, number of fingers; PT, productive tiller.

**Table 4:** The average grain yield (ton ha^−1^) of finger millet genotypes evaluated at each environment.

Genotype	Genotype code	Environments					Mean
		AN18	AN19	AS19	AX18	AX19	
IEFX12015	G1	1.588	1.693	1.364	2.002	1.954	1.720
IEFX12027	G2	1.642	1.981	1.766	1.904	1.940	1.847
IEFX12003	G3	1.794	3.090	2.041	1.862	1.947	2.147
IEFX12017	G4	1.671	2.407	2.163	1.749	1.877	1.973
IEFV 10017	G5	1.419	1.099	1.854	1.685	1.776	1.567
IEFX12035	G6	1.905	3.378	1.663	2.104	2.083	2.227
IEFV 10004	G7	1.587	1.760	1.640	1.899	1.915	1.760
4646	G8	1.638	3.047	2.240	1.570	1.738	2.047
IEFX12024	G9	1.697	1.845	1.282	2.167	2.076	1.813
IEFX12018	G10	1.629	2.335	1.534	1.904	1.899	1.860
IEFX12023	G11	1.583	1.996	1.135	2.022	1.930	1.733
IEFV 10006	G12	1.479	2.105	1.511	1.725	1.747	1.713
IEFX12022	G13	1.716	2.566	1.674	1.950	1.960	1.973
IEFX12011	G14	1.636	2.257	1.937	1.794	1.876	1.900
IEFV 10018	G15	2.022	1.985	2.374	2.270	2.349	2.200
KAT FM1	G16	1.619	3.055	1.944	1.638	1.744	2.000
IEFX12028	G17	1.787	2.761	2.200	1.851	1.967	2.113
IEFX12021	G18	1.607	3.467	1.459	1.713	1.721	1.993
IEFX12026	G19	1.722	3.453	1.542	1.859	1.858	2.087
IEFX12010	G20	1.633	1.952	1.454	1.998	1.964	1.800
6350	G21	1.556	1.422	2.256	1.705	1.861	1.760
IEFX12002	G22	1.540	1.241	1.942	1.813	1.898	1.687
3189	G23	1.731	1.577	2.850	1.745	1.996	1.980
2710	G24	1.521	2.749	2.785	1.265	1.579	1.980
Okhale 1	G25	1.883	3.988	1.876	1.903	1.949	2.320
IEFX12013	G26	1.727	1.237	1.422	2.264	2.184	1.767
IEFX12030	G27	1.552	2.022	1.894	1.722	1.810	1.800
IEFX12034	G28	1.622	2.156	1.739	1.855	1.894	1.853
IEFX12016	G29	1.911	3.22	2.055	2.009	2.072	2.253
IEFX12029	G30	1.747	2.214	1.786	2.017	2.036	1.960
5106	G31	1.436	2.256	1.550	1.623	1.668	1.707
IEFX12005	G32	2.177	4.884	2.361	2.038	2.140	2.720
IEFX12004	G33	1.704	2.833	1.774	1.857	1.898	2.013
KNE796	G34	1.897	3.766	2.174	1.862	1.967	2.333
IEFX12020	G35	1.524	1.299	1.270	2.001	1.939	1.607
IEFX12032	G36	1.515	2.359	1.605	1.707	1.747	1.787
IEFX12019	G37	1.494	2.574	1.659	1.622	1.684	1.807
IEFX12014	G38	1.557	1.846	1.374	1.929	1.894	1.720
3945	G39	1.974	3.888	2.518	1.843	2.01	2.447
IEFX12012	G40	1.629	1.818	2.171	1.777	1.905	1.860
IEFV 10028	G41	1.654	2.526	2.133	1.713	1.841	1.973
6473	G42	2.064	3.948	2.556	1.955	2.110	2.527
IEFV 10005	G43	1.740	2.081	2.352	1.84	1.987	2.000
IEFX12025	G44	1.650	2.953	1.805	1.747	1.811	1.993
IEFV 10027	G45	1.683	1.402	3.03	1.643	1.942	1.940
IEFX12031	G46	1.712	1.950	1.226	2.191	2.087	1.833
IEFX12007	G47	1.751	2.358	1.586	2.064	2.041	1.960
IEFX12008	G48	1.763	2.603	1.728	1.997	2.008	2.020
6059	G49	1.634	1.231	2.389	1.808	1.971	1.807
IEFV 10021	G50	1.634	3.119	1.593	1.765	1.789	1.980
IEFX12009	G51	1.851	2.377	1.139	2.358	2.209	1.987
IEFX12033	G52	1.861	4.233	1.893	1.826	1.887	2.340
IEFX12001	G53	1.721	2.538	1.852	1.904	1.952	1.993
IEFX12036	G54	1.694	1.974	2.197	1.841	1.961	1.933
Tessema (check)	G55	1.848	2.014	1.652	1.758	1.785	1.811
Mean		1.697	2.453	1.872	1.866	1.924	

*Note:* AN18, Negele Arsi 2018; NA19, Negele Arsi 2019; AS19, Assosa 2019; AX18, Axum 2018 and AX19, Axum 2019.

**Table 5:** Analysis of variance of key agronomic traits (mean squares) for 55 finger millet genotypes.

Source of variations	DF	Mean square			
		Finger length	Number of fingers	Plant height	Grain yield
Replication	2	7.05	2.12	3393.3	0.38^∗^
Genotypes	54	5.26^∗∗∗^	3.48^∗∗∗^	526.5ns	0.80^∗∗^
Residual	768	2.08	2.18	438.2	0.49

*Note:* The symbols ^∗∗∗^ and ^∗∗^ indicate highly significant at *p* < 0.001 and significant at *p* = 0.01, respectively. The word ns indicates nonsignificant.

^∗^Significance at *p* = 0.05.

**Table 6:** Analysis of phenotypic and genotypic percentages of key agronomic traits for finger millet genotypes.

Traits	Mean	PCV (%)	GCV (%)	ECV (%)
Finger length	6.49	27.31	15.86	22.24
Number of fingers	6.53	24.74	10.11	22.58
Plant height	91.71	23.58	5.91	22.82
Grain yield	1.96	39.36	16.06	35.94

Abbreviations: ECV, environmental coefficient of variance; GCA, genotypic coefficient of variance; PCV, phenotypic coefficient of variance.

**Table 7:** Stability coefficients of the grain yield for finger millet genotypes that were tested in five environments.

Genotype	Genotype code	Cultivar superiority value	Mean	Rank	Static stability	Rank	Wricke's ecovalence	Rank	Mean ranks value	Rank	MADPR value	Rank	Variance of ranks value	Rank
IEFX12015	G1	1.51	1.72	49.00	0.11	12.00	0.67	27.00	34.50	46.00	20.30	35.50	295.90	36.00
IEFX12027	G2	1.19	1.85	35.00	0.05	5.00	0.22	9.00	31.10	38.00	11.50	6.00	85.90	6.00
IEFX12003	G3	0.59	2.15	9.00	0.32	39.00	0.70	29.00	23.00	10.50	21.80	41.00	327.10	43.00
IEFX12017	G4	0.91	1.97	23.00	0.14	18.00	0.18	4.00	27.80	26.00	14.40	9.50	137.10	9.00
IEFV 10017	G5	1.84	1.57	54.00	0.12	15.00	1.29	42.00	40.40	55.00	18.40	26.00	235.80	24.00
IEFX12035	G6	0.52	2.23	7.00	0.49	45.00	0.73	31.00	17.10	6.00	15.30	16.00	164.00	14.00
IEFV 10004	G7	1.41	1.76	46.00	0.06	6.00	0.75	32.00	36.10	50.00	17.00	19.00	265.30	31.00
4646	G8	0.70	2.05	13.00	0.44	44.00	0.69	28.00	28.30	27.00	21.90	42.00	313.70	40.00
IEFX12024	G9	1.35	1.81	44.00	0.11	14.00	0.73	30.00	28.90	30.00	21.70	40.00	318.40	42.00
IEFX12018	G10	1.05	1.86	30.00	0.11	13.00	0.15	3.00	32.20	41.00	14.90	13.00	143.10	10.00
IEFX12023	G11	1.44	1.73	48.00	0.21	33.00	1.06	36.00	35.00	47.00	23.50	47.00	375.40	45.00
IEFV 10006	G12	1.30	1.71	41.00	0.08	9.00	0.12	2.00	39.20	53.00	9.60	2.00	60.30	2.00
IEFX12022	G13	0.87	1.97	21.00	0.17	22.00	0.25	12.00	26.20	19.00	19.60	31.00	249.70	29.00
IEFX12011	G14	1.04	1.90	28.00	0.17	21.00	0.31	14.00	29.80	36.00	19.50	30.00	238.90	26.00
IEFV 10018	G15	0.96	2.20	24.00	0.19	27.00	1.08	37.00	16.90	4.50	20.00	33.00	300.40	37.00
KAT FM1	G16	0.73	2.00	14.00	0.51	46.00	0.87	34.00	29.20	31.50	23.10	45.00	393.10	46.00
IEFX12028	G17	0.70	2.11	12.00	0.24	34.00	0.27	13.00	21.80	9.00	14.50	11.00	175.60	17.00
IEFX12021	G18	0.69	1.99	11.00	0.71	52.00	1.55	48.00	29.30	33.00	25.30	49.00	413.50	48.00
IEFX12026	G19	0.64	2.09	10.00	0.64	48.00	1.11	39.00	27.50	23.50	18.00	24.00	217.10	22.00
IEFX12010	G20	1.27	1.80	40.00	0.05	4.00	0.35	18.00	33.20	43.00	14.70	12.00	144.30	11.00
6350	G21	1.57	1.76	51.00	0.20	30.00	1.35	44.00	31.40	39.00	27.70	54.00	520.20	54.00
IEFX12002	G22	1.67	1.69	52.00	0.14	17.00	1.22	40.00	35.30	48.00	18.60	27.00	225.00	23.00
3189	G23	1.31	1.98	42.00	0.27	36.00	1.97	52.00	27.30	22.00	27.30	53.00	493.60	53.00
2710	G24	0.85	1.98	19.00	0.52	47.00	1.46	47.00	31.80	40.00	28.20	55.00	521.30	55.00
Okhale 1	G25	0.35	2.32	4.00	0.90	53.00	1.86	50.00	20.00	7.00	18.80	28.00	236.50	25.00
IEFX12013	G26	1.70	1.77	53.00	0.20	28.00	1.86	49.00	27.70	25.00	25.80	50.00	476.30	52.00
IEFX12030	G27	1.21	1.80	36.00	0.16	19.00	0.43	21.00	33.00	42.00	20.70	37.50	269.90	32.00
IEFX12034	G28	1.16	1.85	33.00	0.16	20.00	0.33	16.00	29.20	31.50	19.10	29.00	274.30	33.00
IEFX12016	G29	0.55	2.25	8.00	0.40	40.00	1.09	38.00	23.00	10.50	22.20	43.00	314.60	41.00
IEFX12029	G30	0.99	1.96	25.00	0.04	2.00	0.18	5.00	25.40	16.00	16.00	17.00	166.70	15.00
5106	G31	1.24	1.71	38.00	0.21	32.00	0.24	11.00	39.30	54.00	14.00	8.00	127.30	8.00
IEFX12005	G32	0.12	2.72	1.00	1.46	55.00	3.62	55.00	9.00	1.00	10.20	3.00	68.50	3.00
IEFX12004	G33	0.82	2.01	17.00	0.41	43.00	0.63	25.00	24.30	12.00	19.90	32.00	311.70	39.00
KNE796	G34	0.38	2.33	6.00	0.69	50.00	1.27	41.00	16.30	3.00	15.00	14.00	188.20	18.00
IEFX12020	G35	1.85	1.61	55.00	0.20	29.00	1.38	45.00	37.20	52.00	23.40	46.00	447.10	49.00
IEFX12032	G36	1.17	1.79	34.00	0.17	24.00	0.34	17.00	34.10	45.00	17.30	21.00	203.70	20.00
IEFX12019	G37	1.05	1.81	29.00	0.25	35.00	0.22	10.00	36.30	51.00	15.20	15.00	150.90	12.00
IEFX12014	G38	1.38	1.72	45.00	0.07	8.00	0.47	23.00	36.00	49.00	18.30	25.00	244.90	27.00
3945	G39	0.25	2.45	3.00	0.70	51.00	1.45	46.00	16.90	4.50	17.70	23.00	245.80	28.00
IEFX12012	G40	1.26	1.86	39.00	0.06	7.00	0.60	24.00	28.70	29.00	20.70	37.50	310.10	38.00
IEFV 10028	G41	0.89	1.97	22.00	0.17	23.00	0.21	8.00	26.70	20.00	17.50	22.00	206.10	21.00
6473	G42	0.21	2.53	2.00	0.67	49.00	1.31	43.00	10.30	2.00	10.30	4.00	83.40	4.00
IEFV 10005	G43	1.00	2.00	27.00	0.04	3.00	0.39	20.00	25.20	14.00	14.40	9.50	155.10	13.00
IEFX12025	G44	0.75	1.99	16.00	0.28	37.00	0.33	15.00	29.50	34.50	16.10	18.00	171.90	16.00
IEFV 10027	G45	1.43	1.94	47.00	0.41	41.00	2.49	53.00	30.80	37.00	22.40	44.00	348.10	44.00
IEFX12031	G46	1.32	1.83	43.00	0.18	25.00	0.76	33.00	27.50	23.50	26.00	51.00	458.80	50.00
IEFX12007	G47	1.00	1.96	26.00	0.19	26.00	0.36	19.00	25.30	15.00	21.60	39.00	292.10	35.00
IEFX12008	G48	0.84	2.02	18.00	0.12	16.00	0.20	6.00	25.80	18.00	13.60	7.00	126.70	7.00
6059	G49	1.56	1.81	50.00	0.20	31.00	1.90	51.00	28.40	28.00	26.30	52.00	472.70	51.00
IEFV 10021	G50	0.73	1.98	15.00	0.41	42.00	0.66	26.00	29.50	34.50	20.30	35.50	262.90	30.00
IEFX12009	G51	1.06	1.99	31.00	0.32	38.00	0.99	35.00	24.40	13.00	24.80	48.00	400.20	47.00
IEFX12033	G52	0.36	2.34	5.00	1.13	54.00	2.53	54.00	20.50	8.00	17.20	20.00	189.10	19.00
IEFX12001	G53	0.86	1.99	20.00	0.10	10.00	0.02	1.00	25.70	17.00	4.90	1.00	17.60	1.00
IEFX12036	G54	1.13	1.93	32.00	0.10	11.00	0.47	22.00	26.90	21.00	20.10	34.00	284.70	34.00
Tessema	G55	1.23	1.81	37.00	0.03	1.00	0.21	7.00	33.80	44.00	10.90	5.00	84.00	5.00

*Note:* Rank shows the position of each genotype according to the stability coefficient in the previous column.

Abbreviation: MADPR, mean absolute difference of pairs of ranks.

**Table 8:** AMMI analysis of variance for the grain yield of 55 finger millet genotypes evaluated across locations.

Source of variations	DF	SS	MS	% of variances	Significance
Total	824	425.6	0.517		
Treatments	274	240.8	0.879		^∗∗∗^
Genotypes	54	42.9	0.795	10.08	^∗∗∗^
Environments	4	54.3	13.578	12.76	^∗∗∗^
G x E	216	143.6	0.665	33.74	^∗∗∗^
IPCA 1	57	86.8	1.523		^∗∗∗^
IPCA 2	55	28.3	0.515		^∗∗^
Residuals	104	28.5	0.274		
Error	540	171.3	0.317		

*Note:* The symbols ^∗∗∗^ and ^∗∗^ represent highly significance at < 0.001 and 0.001, respectively.

Abbreviations: DF, degree of freedoms; MS, mean square; SS, sum of square.

## Data Availability

No data were used for the research article.
